# Self-medication practices and associated knowledge among health sciences students: A cross-sectional study

**DOI:** 10.1177/20503121261490603

**Published:** 2026-09-26

**Authors:** Deepshikha Ghimire, Santosh Gaihre, Padma Prasad Khatiwada, Pasang Tamang, Dikshya Khadka, Alisha Ghale, Muhammad Abid, Dev Acharya

**Affiliations:** 1 Faculty of Medicine and Health Sciences, Department of Public Health, 8695 University of Wolverhampton; 2 Institute of Applied Health Sciences, School of Medicine, Medical Sciences and Nutrition, 1019 University of Aberdeen; 3 Central Department of Population Studies, 97983 Tribhuvan University, Nepal; 4 Faculty of Education, Health and Human Sciences, 4918 University of Greenwich; 5 Faculty of Health, Sport and Behavioural Sciences, 6087 University of Northampton

**Keywords:** self-medication, university students, knowledge, prevalence, over-the-counter drugs, health behaviour

## Abstract

**Objectives:**

To investigate the prevalence, knowledge, and factors associated with self-medication among students at the University of Wolverhampton, UK, including examination of medication knowledge, advertising influences, and awareness of harmful effects.

**Methods:**

A cross-sectional study was conducted among health and public health students at the University of Wolverhampton. A pre-tested, structured online questionnaire collected data on demographics, knowledge, attitudes, and self-medication practices. Ethical approval was obtained from the University of Wolverhampton Faculty Ethics Committee, and all participants provided written informed consent prior to their participation in the study. Data were analysed using SPSS, with descriptive statistics presented as frequencies and percentages, and binary logistic regression to examine associations between self-medication and socio-demographic variables (p < 0.05).

**Results:**

A total of 106 students were invited to participate, and 50 completed the questionnaire, yielding a response rate of 47.2%. Reasons for non-participation were not collected, as the anonymous survey design precluded tracking of individual non-respondents. Of 50 respondents, 60% reported practising self-medication in the past three months. Female students (76%) and master’s students (79%) were more likely to engage in self-medication than male students (24%) and bachelor’s students (6%). All participants were aware of self-medication, with 92% recognising dangerous drug interactions and 76% aware of organ damage and other risks. Common sources of information included the internet (86%), parents/relatives (76%), and friends (64%). Pain relievers, cough/cold medications, and antihistamines were the most frequently used drugs, primarily for minor illnesses such as headaches and colds/flu. Binary logistic regression showed significant associations between gender and year of study with self-medication prevalence.

**Conclusion:**

Self-medication is widespread among university students, despite high awareness of its potential risks. Female and master’s students are particularly prone to self-medication. These findings highlight the need for targeted educational interventions, university-based health promotion strategies, and stricter over-the-counter medicine regulations to ensure responsible self-medication. Future research should include larger, multicentre studies and explore cultural influences on self-medication behaviours.

## Introduction

Self-medication involves procuring and ingesting medicines to treat self-recognised ailments without consulting healthcare professionals.^1^ Globally, self-medication is influenced by age, gender, income, health literacy, and access to pharmacies.^2,3^ The irrational use of antibiotics without a doctor’s prescription has risen tremendously, exceeding 50% globally, and is prevalent in developed, middle-income, and developing countries.^2,3^ A global meta-analysis^4^ found that university students exhibited the highest prevalence of self-medication during the pandemic (54.5%), significantly exceeding the rates found in healthcare workers. Studies indicate self-medication prevalence of 94.5% among adolescents in the Middle East^5^ In the UK and Ireland, while media reports suggest a surge in ‘smart drug’ use, a large-scale survey found that the lifetime prevalence of prescription stimulants for cognitive enhancement remains under 10%, with regular use being as low as 0.3-4%^6^ However, there is a substantial interest in these substances, with approximately one-third of students expressing curiosity about their use [^6^].

University students are particularly susceptible due to educational qualities, access to information via social platforms, and pharmacy accessibility.^7,8^ The inappropriate use of painkillers, dietary supplements, and tranquilisers has been reported.^9^ Educated individuals are more likely to understand risks, yet public knowledge and effective interventions are needed to reduce antibiotic misuse and self-medication practices. To our knowledge, no study has examined self-medication among University of Wolverhampton students, within a specific, international-heavy student population in the West Midlands.

## Methods

### Study design and duration

This study was a **faculty-based** cross-sectional survey conducted specifically within the Faculty of Health, Education and Wellbeing at the University of Wolverhampton. While the findings provide localised insights into this faculty, they are not intended to represent the university-wide student body. This cross-sectional study is reported in accordance with the Strengthening the Reporting of Observational Studies in Epidemiology (STROBE) guideline for cross-sectional studies^10^ Data collection was carried out over three months, from October to December 2023. The total research process, including preparation, questionnaire development, pretesting, data collection, and analysis, spanned approximately six months.

### Study population

The study population consisted of students enrolled in health-related modules. The survey was open to eligible students within the Faculty of Health, Education and Wellbeing, although the final sample predominantly comprised postgraduate students. The final sample ( n =50) was overwhelmingly composed of postgraduate (Master’s) students (86%, n =43), with minimal representation from undergraduate students (6%, n =3). Consequently, this study is treated as an exploratory investigation primarily focusing on the postgraduate subgroup, and the undergraduate data are interpreted with caution due to the small sub-sample size.

Students were eligible to participate if they were aged 18 years or above and were currently enrolled in a health or public health program. Exclusion criteria included students under 18 years of age (due to inability to provide legal informed consent), students not enrolled in health-related programmes (as they were not relevant to the study objectives), and incomplete questionnaire responses (to ensure data validity and reliability). The target population included students from different years of study, living arrangements, and educational backgrounds, which allows a comprehensive understanding of self-medication practices within this group.

### Study setting

The study was conducted within the Faculty of Health, Education and Wellbeing at the University of Wolverhampton, located in the West Midlands, UK. This institution features a diverse student body with a significant international population across various academic levels. The research specifically targeted students enrolled in the faculty’s health and public health programs, with data collection occurring between October and December 2023.

### Sample size and sampling technique

The initial target sample size was 106 students, calculated using Cochran’s formula^11^ and accounting for a 10% non-response rate to ensure the study remained adequately powered.^12,13^ This lower response rate is acknowledged as a limitation and may impact the generalisability of the findings. Furthermore, the small sample size may introduce non-response bias, as those who chose to participate might have a higher interest in, or more frequent practice of, self-medication than those who did not. Convenience sampling^14^ was adopted to facilitate data collection within the three-month window. This approach introduces potential selection bias, as students with a higher personal interest in self-medication may have been more likely to respond to the invitation.

While the final sample ( n =50) is below the initial target for a full-scale survey, it meets the established criteria for an exploratory study, where a sample size of 30–50 is generally considered sufficient to assess the reliability of a research instrument and identify primary behavioural patterns within a specific subgroup.^15^ Therefore, while not intended to provide definitive university-wide evidence, these findings serve as an essential preliminary assessment of international student health behaviours. As shown in Figure 1, the participant recruitment and enrollment process illustrates the flow of eligible students through the study.

**Figure 1.**
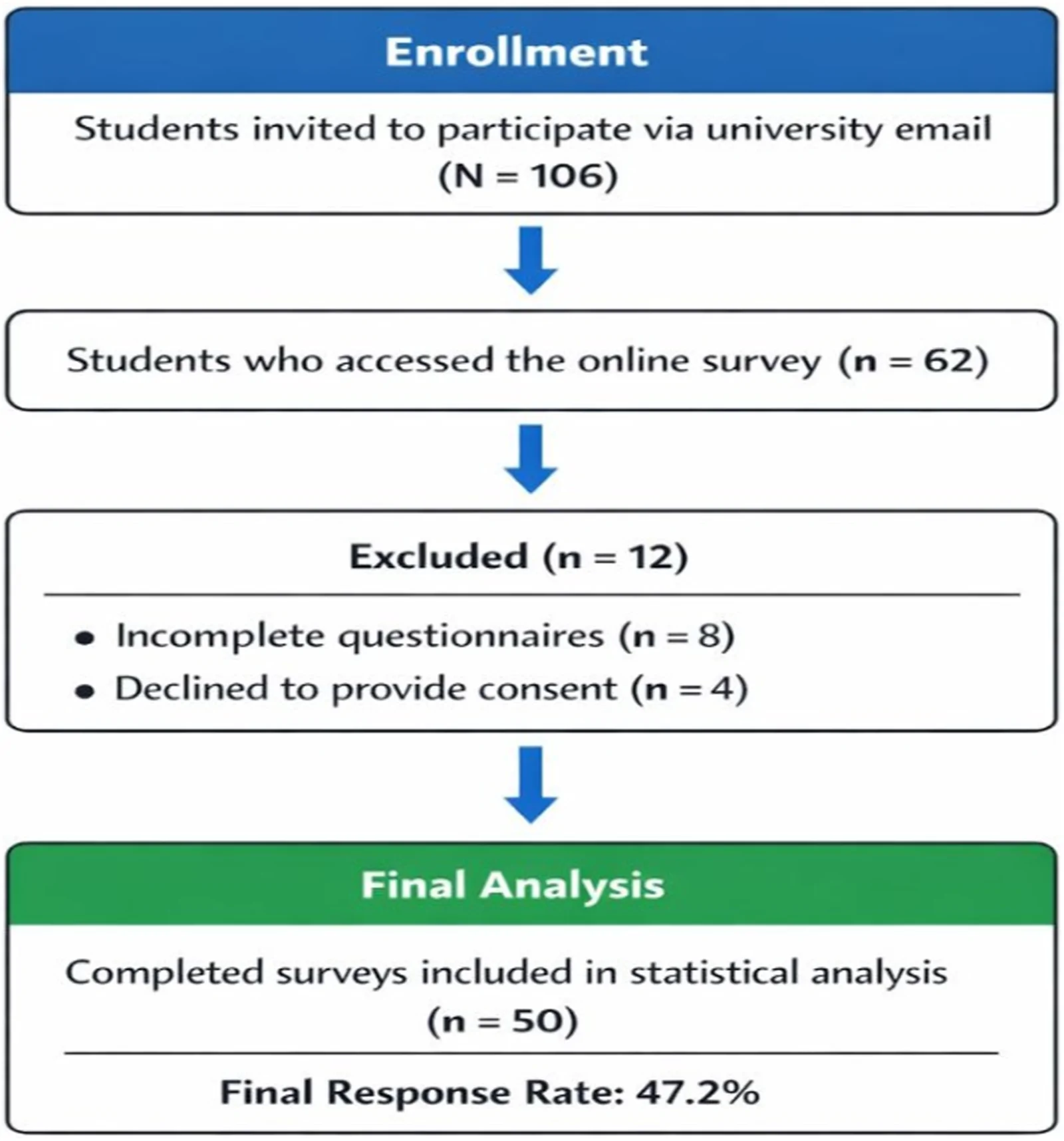
Flow diagram of participant recruitment and study enrollment.

### Study tool and procedure

A structured, anonymous questionnaire was designed using Google Forms, informed by literature and guidelines on health survey design^16^ and questionnaire development.^17^ Data collection was conducted via this online platform to ensure student safety and accessibility. While online surveys can sometimes result in lower response rates compared to in-person distribution, this method was chosen to facilitate participation among a diverse, faculty-wide student body with varying schedules.

The questionnaire was reviewed by the research supervisor and subject experts to establish face and content validity, ensuring the items accurately reflected the study’s objectives.^18^ A pre-test was conducted with 10% of the target sample (the pre-test phase) to assess clarity, relevance, and instrument reliability.^19^ During this pre-testing phase, the questionnaire was evaluated for comprehensibility and logical flow. However, as the survey consisted primarily of independent categorical items and multiple-response questions, rather than Likert-scale constructs, a formal Cronbach’s alpha was not calculated. Feedback from the pre-test phase

Led to minor modifications to improve wording and participant understanding before the final deployment. The questionnaire included both open and closed-ended questions and was divided into five sections.

• **Socio-demographic details:** Age, sex, year of study, and living status.
• **Knowledge of self-medication:** Awareness, sources of information, understanding of over-the-counter drugs, benefits, risks, and side effects.
• **Self-medication practices:** Frequency, conditions prompting self-medication, types of drugs used, and sources of recommendation.
• **Attitudes toward self-medication:** Reasons for self-medication, discontinuation, dosage knowledge, and personal experiences.
• **Side effects and cessation:** Experiences of adverse reactions and criteria for stopping self-medication.

### Data collection

The questionnaire was distributed via university email to eligible students. As participation was voluntary and based on accessibility, a non-probability convenience sampling approach was utilised. Before participating, students were provided with information about the study, including objectives, procedures, voluntary participation, and the right to withdraw at any time. To minimise social desirability bias, the questionnaire was anonymous and self-administered, and participants were assured of confidentiality.^18,20^ Unique identifiers were used to maintain anonymity, and no personal identifiers were collected. Access to the form was restricted to prevent duplicate submissions.

### Data analysis

Collected data were analysed using SPSS (version 22.0).^21,22^ Descriptive statistics (frequencies and percentages) were used to summarise socio-demographic characteristics, knowledge, practices, and attitudes. Binary logistic regression^23^ was employed to explore associations between self-medication and independent variables such as age, sex, living status, and year of study. The p-value, odds ratio, and 95% confidence interval were reported. A p-value of less than 0.05 was considered statistically significant. Bar graphs, and pie charts were used to visualise prevalence, common medications, and levels of awareness among participants. Missing data were minimal and handled by complete case analysis, as no variable had more than 5% missing responses.

### Ethical considerations

Ethical approval was obtained from the University of Wolverhampton Faculty Ethics Committee (2320791-/01/08/2023), and all participants provided written informed consent prior to their participation in the study. Participation was voluntary, and students could withdraw at any point without penalty. Data were anonymised and securely stored for research purposes only. All study procedures adhered to ethical principles, including minimising harm, respecting participant’s rights, and ensuring transparency.^24,25^ All sources of information were accurately cited to avoid plagiarism.^26^

## Results

### Demographic and socio-economic characteristics

Table 1 below shows the sociodemographic characteristics of the study respondents. Among 50 participants, 38 (76%) were female and 12 (24%) were male (Figure 2 demonstrates the gender distribution among respondents). The age distribution of the participants is illustrated in Table 1. Only 2 respondents (4%) were from the UK, whereas 48 (96%) were international students. Most respondents were international students, as shown in Table 1. The majority were master’s students (43, 86%), followed by others (4, 8%) and bachelor’s students (3, 6%).

**Figure 2.**
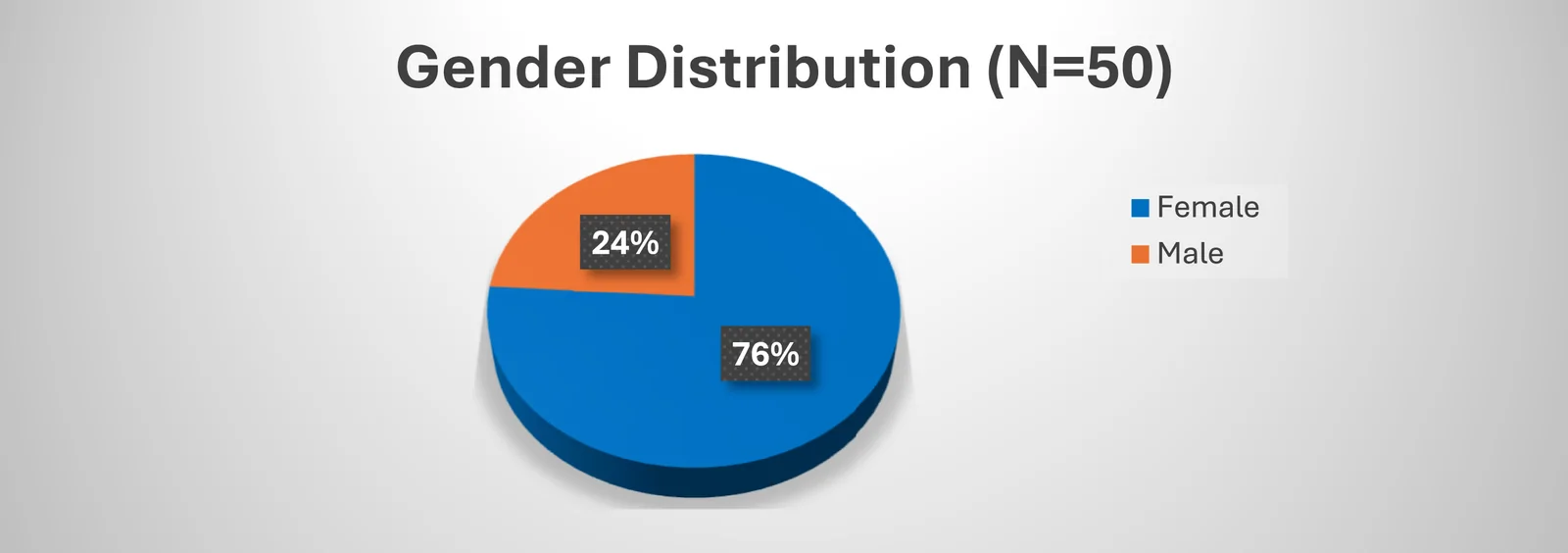
Pie chart showing female (76%) and male (24%).

**Table 1.** Socio-demographic characteristics of the study respondents (n=50).

Demographic variables	Frequency ( n =50)	Percentage (%)
Age (Years)	​	​
Mean ± SD	28.4 ± 3.5	​
Range (Min–Max)	21 – 38	​
Age Group	​	​
20–29 years	31	62%
30–39 years	19	38%
Sex	​	​
Male	12	24%
Female	38	76%
Country of Origin	​	​
United Kingdom	2	4%
International	48	96%
Year of Study	​	​
Bachelor’s	3	6%
Master’s	43	86%
Others	4	8%

### Awareness and knowledge regarding self-medication

All respondents (50, 100%) reported awareness of self-medication (Table 2). Participant sources of information are shown in Figure 3, which illustrates the main sources of knowledge on self-medication, while Table 3 summarises student perceptions regarding the risks of self-medication, such as the potential for harm and delayed treatment. Regarding understanding, 41 (82%) defined it as taking medicine on their initiative, 4 (8%) as taking medicine according to a doctor’s prescription, 11 (22%) based on a previous doctor’s prescription, and 33 (66%) for common health problems without a prescription.

**Table 2.** Knowledge of self-medication (N=50).

Knowledge variables		Total respondents (n)
Have you ever heard about self-medication?	Yes	50
	No	​
What do you understand by self-medication?	Taking medicine on one’s initiative	41
	Taking medicine according to doctor’s prescription	4
	Taking medicine on the doctor’s previous prescription	11
	Taking medicine for common health problems without a doctor’s prescription through pharmacies	33
Source of information about drug for self-medication	Parents/Relatives	38
	Friends	32
	Books	23
	Internet	43
Indications of self-medication	Headaches	48
	Common Cold/Flu	37
	Abdominal problems	32
	Body aches	37
	Chronic Conditions	9
	Other	8
Adverse consequences of self-medication	Organ damage & death	38
	Dangerous drug interaction	46
	Economical loss due to delay in diagnosis and treatment	18
	Others	​

**Figure 3.**
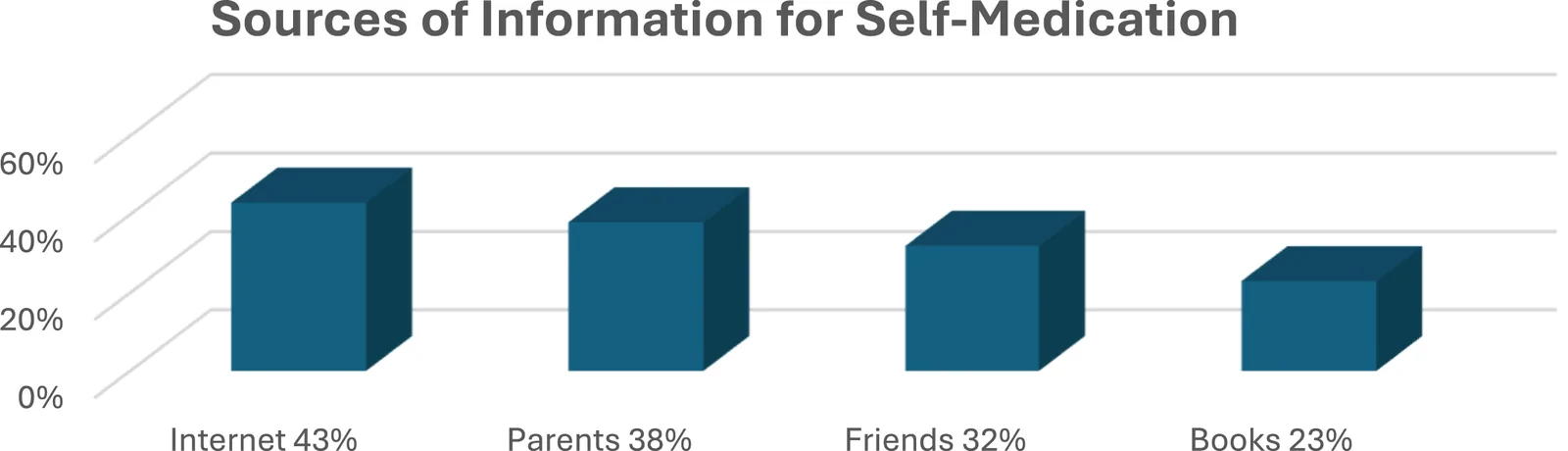
Sources of information for self-medication: Internet (43%), parents (38%), friends (32%), books (23%).

**Table 3.** Students’ perceptions and attitudes toward self-medication risks and drivers ( N =50). Scale: 1 = Strongly disagree, 2 = Disagree, 3 = Neutral, 4 = Agree, 5 = Strongly agree.

Students’ perception of self-medication variables		Total respondents (n)
Practicing self-medication is fruitful	Strongly Agree	4
	Agree	10
	Neutral	13
	Strongly Disagree	13
	Disagree	9
Accessibility of pharmacies everywhere	Strongly Agree	10
	Agree	19
	Neutral	13
	Strongly Disagree	1
	Disagree	6
Advertising medicine encourages self-medication	Strongly Agree	12
	Agree	21
	Neutral	9
	Strongly Disagree	2
	Disagree	5
Home-made remedies	Strongly Agree	18
	Agree	21
	Neutral	5
	Strongly Disagree	3
	Disagree	4
Encountering similar illness after a certain time	Strongly Agree	16
	Agree	22
	Neutral	10
	Strongly Disagree	0
	Disagree	2
Can harm and delay treatments	Strongly Agree	22
	Agree	19
	Neutral	6
	Strongly Disagree	1
	Disagree	2
Global challenges and strong legislation must be implemented	Strongly Agree	21
	Agree	18
	Neutral	9
	Strongly Disagree	1
	Disagree	2

The main sources of information were the Internet (43, 86%), parents/relatives (38, 76%), friends (32, 64%), and books (23, 46%). Headache was the most cited indication (48, 96%), followed by common cold/flu (37, 74%), body aches (37, 74%), and abdominal problems (32, 64%). Chronic conditions and other indications were less frequently mentioned (9, 18%; 8, 16%). Regarding adverse consequences, 46 (92%) recognized dangerous drug interactions, 38 (76%) cited organ damage or death, and 18 (36%) reported economic loss due to delayed treatment.

### Practice of self-medication

30 students (60%) reported practicing self-medication in the past three months. The distribution of medication categories utilised by respondents is illustrated in Figure 4, which demonstrates the types of medicines used for self-medication. Commonly used medicines included pain relievers (28, 56%), cough/cold medications (21, 42%), antihistamines (14, 28%), first-aid products (14, 28%), skin care products and dietary supplements (8, 16%).

**Figure 4.**
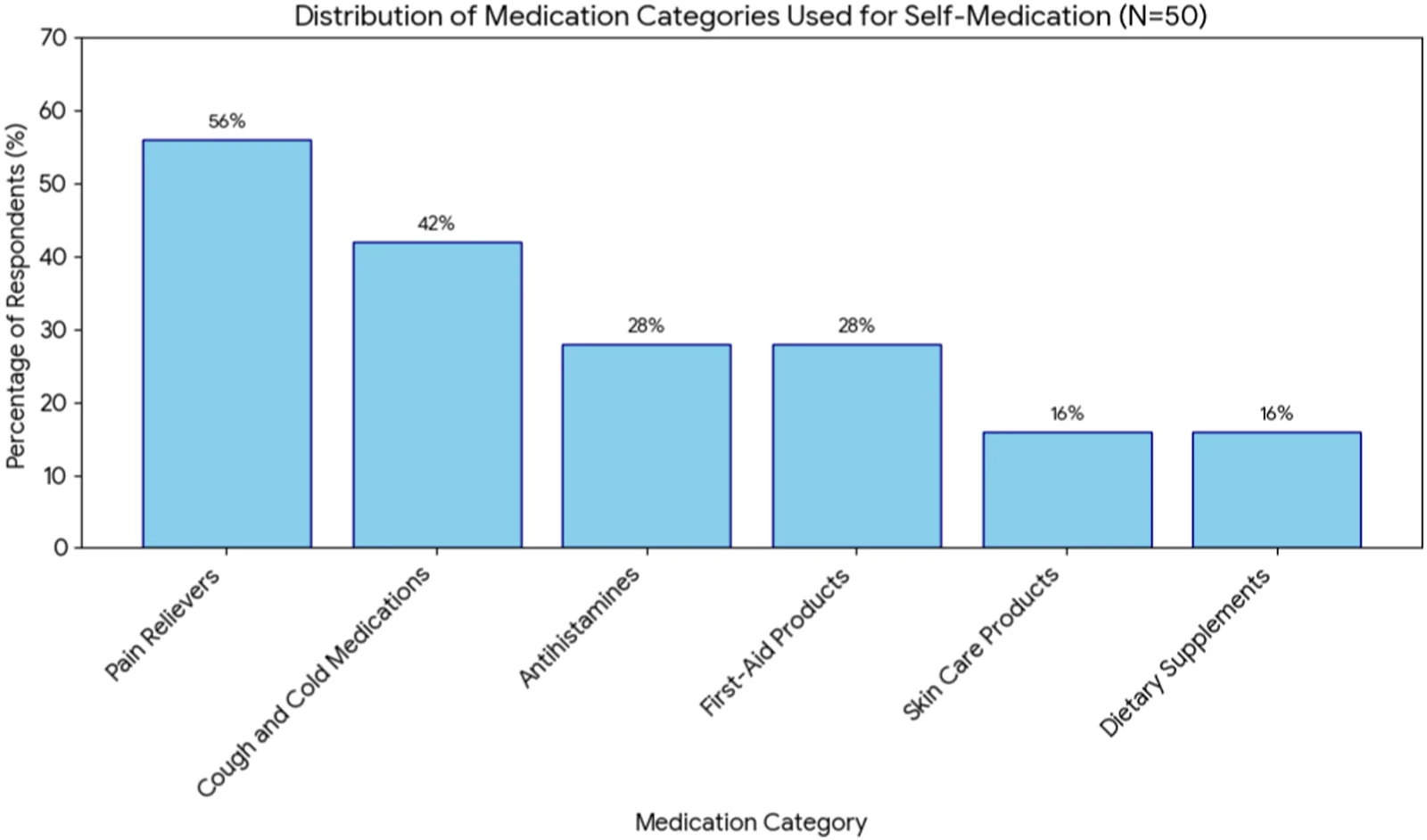
Distribution of medication categories used by respondents for self-medication.

### Attitudes toward self-medication

Among respondents, 14 (28%) reported illness in the last three months, and 30 (60%) practiced self-medication during the same period (Table 4). The specific symptoms prompting these practices are visualised in Figure 5, which shows the health conditions leading to self-medication. Reasons for stopping included risk of wrong diagnosis (28, 56%) and adverse drug reactions (22, 44%). Most participants practiced self-medication seasonally (28, 56%), primarily for minor illnesses (31, 62%), using pain relievers (28, 56%) and cough/cold medication (21, 42%).

**Table 4.** Attitudes toward self-medication ( N =50).

Students’ attitudes of self-medication variables		Total respondents (n)
Do you have any illnesses in the last three months?	Yes	14
	No	36
Have you practiced self-medication in the last three months?	Yes	30
	No	16
	Maybe	4
What was the reason for stopping self-edication?	Risk of adverse drug reaction	22
	Risk of using the wrong diagnosis	28
	Risk of missing actual diagnosis	15
	Risk of drug dependence	20
	Once the symptoms abate	20
How often do you practice self-medication?	Daily	1
	Weekly	0
	Monthly	8
	Seasonal	28
	Yearly	7
	Never	4
What was the reason for being in favor of self-medication?	Lack of time	11
	Minor illness	31
	Good knowledge of pharmacology	23
	Cost-effectiveness	18
	Increased accessibility	14
	Prompt Alleviation	14
What did you know about the medicine that you used for self-medication?	Content	1
	Content and Dose	27
	Content, Dose, and Duration of Therapy	23
	Content, Dose, Duration of therapy, and adverse drug reaction	16
Which of the drugs did you use for self-medication?	Pain Relievers	28
	Antihistamines	14
	Cough and Cold Medications	21
	Skin Care Products	8
	Dietary Supplements	8
	First-Aid Products	14
When do you normally stop taking medicines when you are on self-medication?	After symptoms disappear	33
	After a few days of recovery	20
	After completion of the course	28
	After symptoms reduce	5

**Figure 5.**
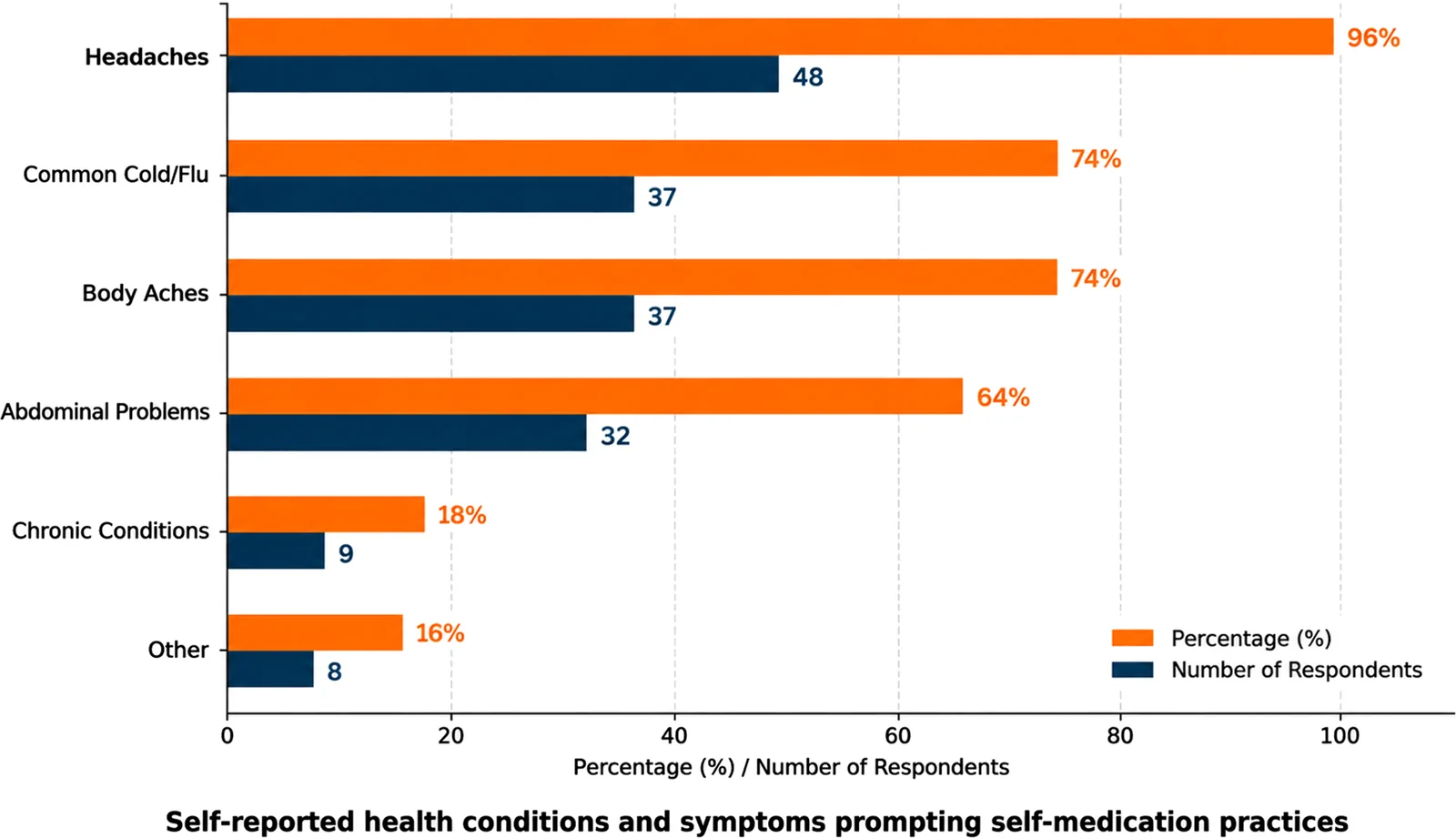
Self-reported health conditions and symptoms prompting self-medication practices.

### Binary logistic regression

Binary logistic regression analysis provided insights into the socio-demographic factors influencing self-medication prevalence (Table 5). However, given the highly skewed nature of the sample (96% international and 86% master’s students), these associations, particularly regarding gender and year of study, must be viewed as preliminary and exploratory. The small number of participants in comparison groups (e.g., n =3 for bachelor’s students) limits the statistical power of these tests, and the findings are not intended to establish definitive causal links but to highlight trends for future large-scale validation.

**Table 5.** Binary Logistics Regression of Demographic, Socio-economic, and self-medication prevalence.

Variable	Category	OR	95% CI	p-value
Sex	Male vs Female	0.146	0.032–0.674	0.014
Country of origin	Others vs UK	1.234	0.114–1.336	0.863
Year of study	Masters’ vs Bachelors	1.502	1.18 – 1.92	0.002

Gender was a significant predictor (p=0.014), with males showing lower odds of practicing self-medication (OR=0.146, 95% CI: 0.032–0.674) compared to females. While the UK-origin participants ( n =2) reported 100% prevalence, the association with country of origin was not statistically significant (p=0.863, OR=1.234, 95% CI: 0.114–1.336). The year of study emerged as another significant predictor, with master’s students more likely to practice self-medication (79.1%) compared to bachelor’s students (33.3%), reflecting higher confidence and autonomy in medication use among more advanced students (p=0.002, OR=1.502, 95% CI: 1.18–1.92). However, given the small number of undergraduate participants ( n =3) and the predominance of international students (96%), these comparative results are exploratory. The study does not definitively differentiate between these subgroups but rather identifies preliminary trends within this specific exploratory cohort.

## Discussion

While self-medication practices among university students have been documented globally, there is limited data concerning international student’s transition into the UK healthcare environment. Unlike existing single-setting studies that focus on domestic students, our findings capture the unique intersection of international health backgrounds and the navigation of a new healthcare system (the NHS). This makes the study more than a mere replication, it is an investigation into the ‘acculturation’ of health behaviours among migrant student populations. This exploratory study addresses this gap by focusing on a diverse cohort at the University of Wolverhampton. While this is an exploratory study with a small sample size ( n =50), it offers unique novelty by focusing on a 96% international student population. While the sample included only two UK-origin students, preventing a robust statistical comparison between domestic and international cohorts, this homogeneity allowed for a focused examination of international postgraduate needs, which is a significant yet under-researched demographic in UK health-seeking literature. This provides rare insights into how migrant students navigate the UK healthcare system, a demographic often overlooked in existing self-medication literature.

Understanding the knowledge, attitudes, and practices of university students regarding self-medication is vital for designing effective health education strategies and ensuring the safe use of medications. This study explored the prevalence, determinants, and perceptions of self-medication among health and public health students at the University of Wolverhampton, UK. The demographic profile of the respondents indicates many female students (76%), most being international students (96%), and a predominant enrolment in master’s programs (86%), which aligns with similar studies examining self-medication among student populations.^7–9^ This high prevalence of international participants suggests that self-medication behaviours may be influenced by health practices in their countries of origin, where over-the-counter access to medications often differs significantly from UK regulations. In this study, students from diverse backgrounds showed varying self-medication behaviours. This aligns with^4^ who noted a geographical disparity in self-medication prevalence, with lower rates in Europe (40.8%) compared to Asia (53%). For international students at Wolverhampton, this suggests a process of ‘health acculturation’ where students may be transitioning from high-prevalence environments to the more regulated UK healthcare system. Furthermore, the higher representation of females may reflect both the demographic composition of health and public health programs and a greater willingness to participate in health-related surveys.

The findings reveal that all respondents (100%) were aware of self-medication, indicating a high level of knowledge. Our findings align with recent 2024-2025 studies^27^ which indicate that university students remain a high-risk group for self-medication due to academic stress and easy access to digital health information. This confirms that the trends observed in our exploratory study reflect an ongoing global public health challenge. In this an exploratory sample, most respondents (82%) understood self-medication as taking medicine on their own initiative, while 66% reported obtaining medications for common health problems without consulting a doctor. Sources of information were primarily the Internet (86%), parents/relatives (76%), and friends (64%), highlighting the diverse channels through which students acquire knowledge about medications. This heavy reliance on digital sources aligns with the findings^28^ who’s large-scale 2025 study found that individuals in the United Kingdom report significantly higher digital health literacy (β=2.1) than those in other European nations. This elevated digital confidence likely empowers students at the University of Wolverhampton to bypass traditional clinical consultations in favour of self-directed digital health management.^28^ This high digital literacy may lead to ‘over-confidence,’ where students feel equipped to self-diagnose using online resources rather than seeking professional medical advice. Furthermore, awareness of potential adverse consequences was relatively high, with 92% recognising dangerous drug interactions and 76% acknowledging organ damage and death as possible outcomes of self-medication. These results suggest that students are informed of the risks, yet the practice remains widespread, reflecting the global pattern of knowledge not always translating into safer behaviour.^2,3^

In terms of practice, the prevalence of self-medication among respondents was substantial, with 60% reporting engagement in self-medication in the past three months. Seasonal self-medication was the most common (56%), followed by monthly practices (16%), while a small fraction (8%) never practiced self-medication. The types of medications most frequently used included pain relievers (56%), cough and cold medications (42%), antihistamines (28%), first-aid products (28%), and skin care products and dietary supplements (16% each) (Figure 4). These patterns are consistent with prior studies, indicating that minor health issues such as headaches, colds, and body aches drive most self-medication behaviours.^6,9^ Students often stopped medications either after symptoms disappeared (66%) or after completing the prescribed course (56%), reflecting partial adherence to safe practices.

Perceptions and attitudes toward self-medication varied considerably. While a minority strongly agreed that self-medication is fruitful (7.5%), a larger proportion agreed (20.8%), with many taking neutral or negative stances (26.4% strongly disagreed, 17% disagreed). Accessibility of pharmacies was acknowledged as an important facilitator, with 58.5% agreeing or strongly agreeing to the availability of medications. Additionally, 66% of students strongly agreed or agreed that homemade remedies were useful, suggesting that traditional or non-prescription approaches are integrated into self-medication behaviours. A substantial portion recognized potential harm, with 81.1% agreeing or strongly agreeing that self-medication can delay treatment and cause harm, highlighting a conflict between awareness of risks and actual practice. The influence of medicine advertisements was also significant, with 66% agreeing or strongly agreeing that such promotions encourage self-medication, reflecting the impact of marketing on student behaviour.

The data also highlight the motivations and barriers associated with self-medication. Most respondents favoured self-medication for minor illnesses (62%), cost-effectiveness (36%), and prompt alleviation of symptoms (28%). Conversely, reasons for discontinuing self-medication included concerns over misdiagnosis (56%), adverse drug reactions (44%), and drug dependence (40%). Figure 5 illustrates the key indications for self-medication, with headaches (96%) and common cold/flu (74%) being the most prevalent conditions, while Table 2 demonstrates knowledge levels regarding the correct approach to self-medication.

Overall, while knowledge about self-medication and its risks is relatively high among students, this has not necessarily translated into safer practices. The high prevalence of self-medication, despite awareness of potential harm, underscores a gap between knowledge and behaviour, consistent with findings from other student populations globally.^2,9^ The high level of homogeneity in this an exploratory sample, being largely comprised of international postgraduates, limits the ability to perform a robust comparative analysis across different nationalities or academic levels. Consequently, the study does not aim to definitively differentiate behaviours between these subgroups. Instead, it highlights a critical ‘knowledge-practice gap’ within this specific cohort, despite 100% awareness of self-medication and high recognition of its risks (92%), 60% of participants still engaged in the practice. This suggests that for international postgraduate students, academic level and health knowledge are not sufficient barriers to prevent self-medication.

From a theoretical perspective, this study contributes to the understanding of ‘health acculturation.^29,30^ According to the framework discussed by,^29^ the transition into a host culture involves specific acculturation strategies, such as marginalisation, which can create stressors that drive individuals toward alternative health behaviours like self-medication. While previous replicative studies focus on domestic student populations, our findings highlight how international students, predominantly from regions where over-the-counter access to medications is less regulated, navigate the transition into the UK’s more restrictive healthcare environment. This ‘behavioural carry-over’^31^ suggests that health education must go beyond simple risk awareness and address the structural and cultural differences in healthcare access that international students encounter upon arrival. The role of accessibility, cultural perceptions, and academic level appears to significantly influence these behaviours, necessitating targeted interventions to promote safe medication practices.

## Strengths and limitations

The primary strength of this study is its focus on university students in health and public health programs, offering a targeted understanding of a population with both academic knowledge and practical engagement in health behaviours. However, as this is an exploratory study with a small sample size ( n =50), the findings should be interpreted with caution and may not be representative of the entire student body. Additionally, the use of structured questionnaires and binary logistic regression provides rigorous quantitative insights into associations between socio-demographic factors and self-medication practices. Visual tools such as bar graphs, flow diagrams and pie charts further aid the clarity of results presentation.

However, limitations must be acknowledged. The study relies on self-reported data, introducing the potential for social desirability and recall bias. Efforts to minimise social desirability bias included anonymous questionnaires and assurances of confidentiality.^32^ The sample size is relatively small ( n =50), representing a 47.2% response rate from the original target of 106, and the single-university setting limits generalisability. This may introduce non-response bias, as students who did not participate might have different self-medication habits. The predominance of international postgraduate students significantly impacts the study’s external validity, as the findings may not be generalisable to domestic or undergraduate populations. While this skew allows for a deep dive into the international student experience, it makes subgroup comparisons (such as academic level or nationality) less reliable. This is explicitly acknowledged by treating the study as an exploratory investigation. However, as a cross-sectional study, these 50 responses provide a foundational understanding and demonstrate the feasibility of the survey instrument for larger, future research. While this response rate is acknowledged as a limitation, the achieved sample is sufficient for an exploratory study to identify primary trends and inform the design of larger, multicentre future investigations. Age variability was restricted as all participants were over 18, which may not reflect the experiences of younger students. Moreover, the cross-sectional design precludes causal inferences, and further qualitative studies would provide deeper insights into motivations, barriers, and cultural influences impacting self-medication practices. We acknowledge that the small sample size limits the ‘evidence-based’ weight of the findings for broad policymaking. However, as an exploratory investigation, it provides the first localised data on this specific cohort, acting as a catalyst for larger, more robust investigations.

In conclusion, this study highlights the widespread prevalence of self-medication among health and public health students, despite a strong awareness of potential risks. Within this as an exploratory sample, female and master’s students appeared particularly prone to self-medication. The findings emphasise the need for structured educational interventions, robust legislation on over-the-counter medicine use, and university-based health programs to ensure responsible self-medication practices. While students demonstrate knowledge of medication use, adverse effects, and proper cessation practices, these are not consistently reflected in behaviour.

## Conclusion and recommendations

This exploratory study suggests that self-medication is common among the sampled health students at the University of Wolverhampton, female students and master’s-level students appeared more frequently within this exploratory sample to engage in this practice within this specific cohort. Students often rely on pharmacies, online sources, prior prescriptions, and advice from family or friends. Pain relievers and cough/cold medications were the most used drugs, mainly for minor illnesses like headaches and colds. Although this study is exploratory and includes a small sample ( n =50), it provides preliminary insights into student’s knowledge, attitudes, and self-medication practices. The findings show that while students are aware of potential risks, such as dangerous drug interactions and organ damage, this awareness does not always translate into safe practices.

Given these preliminary findings, there is a suggested need for targeted educational programs and awareness campaigns at universities to encourage responsible self-medication. Universities might consider implementing these targeted educational programs and exploring stricter OTC regulations to reduce unsafe self-medication practices. Strengthening guidance on over-the-counter medicine sales and medical advertising may also help protect student health. Future research is essential and should involve larger and more diverse student populations, explore cultural influences on self-medication (especially among international students), and use qualitative methods to better understand motivations and barriers. Overall, further exploration of the collaboration between universities, healthcare professionals, and policymakers is important to promote safe medication practices and protect public health.

## Data Availability

The data supporting this study are available from the corresponding author upon reasonable request.*
